# Supplementary material for: Enhancing Photosynthetic Carbon Transport in Rice Plant Optimizes Rhizosphere Bacterial Community in Saline Soil
Source: Int J Mol Sci. 2024 Nov 13;25(22):12184. doi: 10.3390/ijms252212184 (PMC11594718; doi:10.3390/ijms252212184)
Supplement: Supplementary file 1 [file ijms-25-12184-s001.zip › Supplementary Figures.pdf]

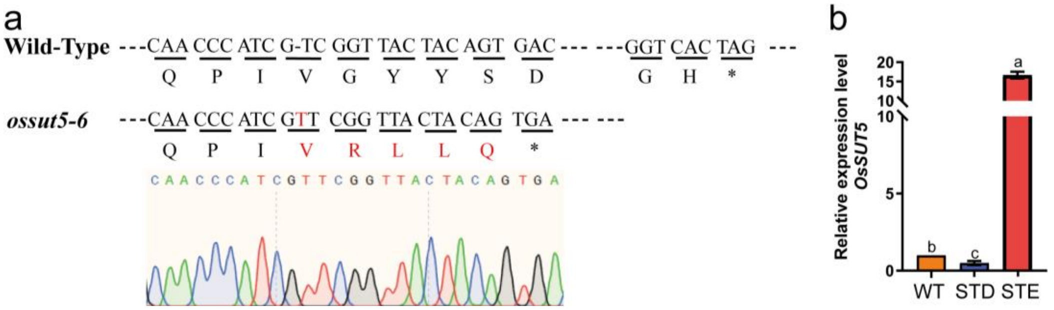

Fig. S1. Verification and phenotype of *ossut5* and OE plants. a, Two lines of *ossut5* plant (STD) were lost due to early termination of protein translation, respectively, caused by a single nucleotide insertion and deletion at the 1st/3rd exon; b, relative expression level of OsSUT5 of WT, STD, and STE rice plants. Mean values with different letters were significantly different ($P < 0.05$).

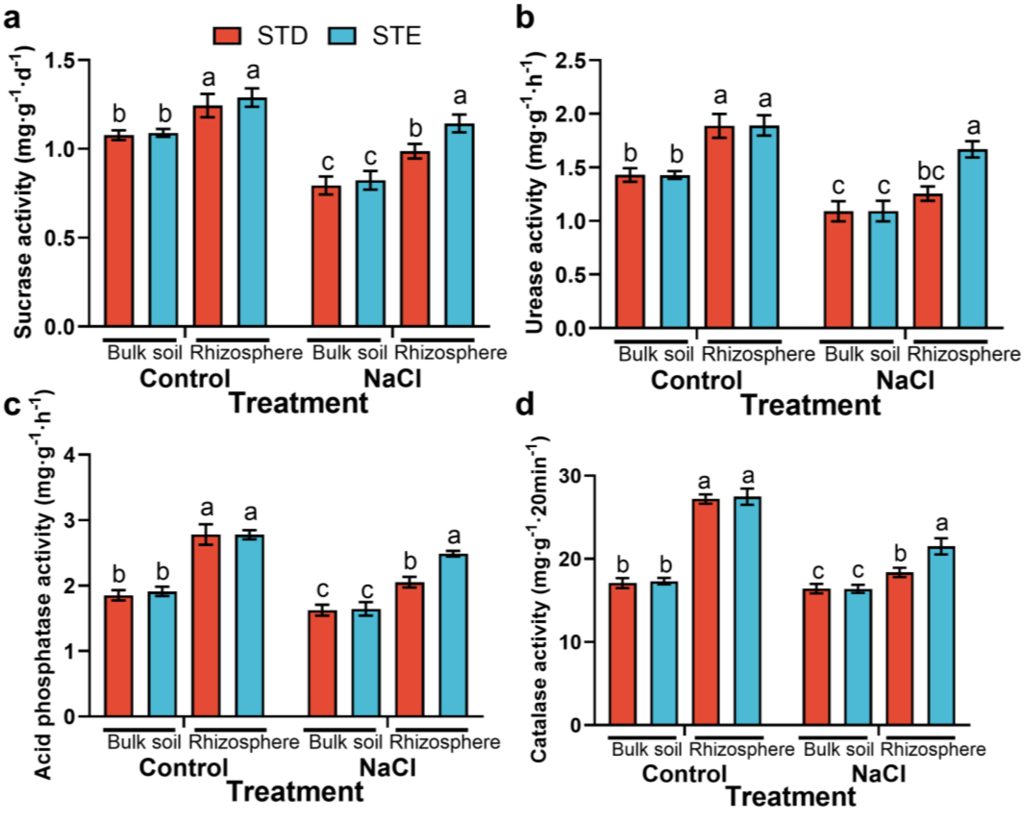

Fig. S2. Sucrose transport engineering affects soil enzyme activities. Soil enzymatic activities of sucrase (a), urease (b), acid phosphatase (c), and catalase (d) were determined with bulk and rhizosphere soils of native, STD, or STE plants at the tillering

stage. The means and standard deviation of duplicate samples were plotted (n = 6). Mean values with different letters were significantly different ($P < 0.05$).

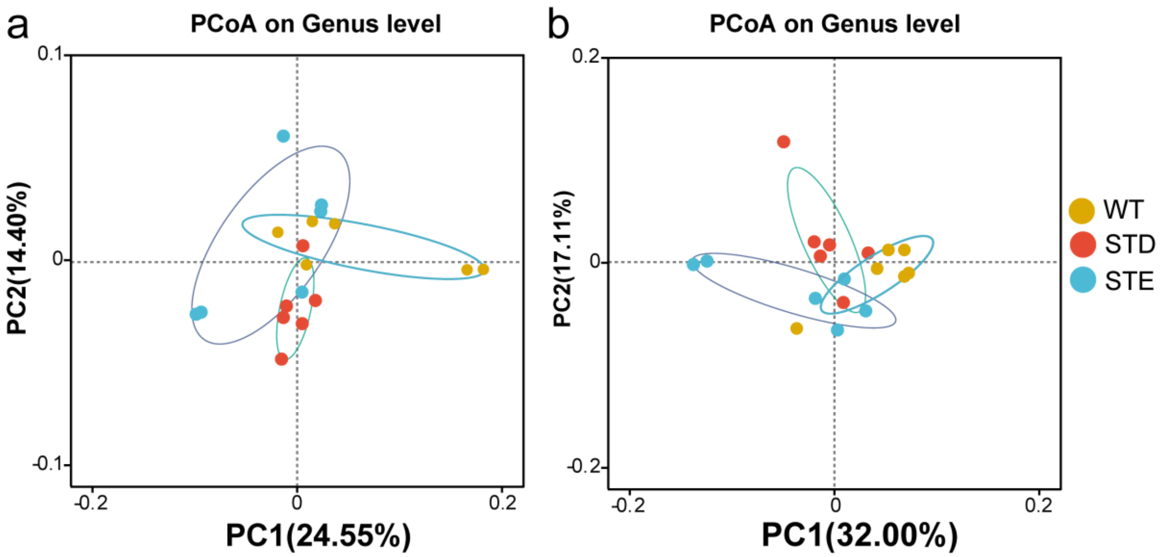

Fig. S3. Principal coordinate analysis (PCoA) ordinations of the bacterial composition of bulk soil under normal or saline conditions. The principal coordinate analysis (PCoA) shows microbial community dissimilarity (Bray–Curtis distance) among bulk soil samples from the three different sucrose transporting circumstances under control (a, R^2=0.3769, P=0.003) or salt stress (b, R^2=0.3093, P=0.003).

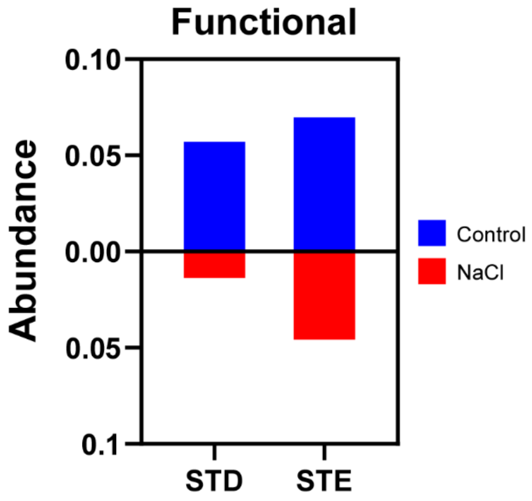

Fig. S4. Functional microbes' response to carbon deposition conditions. Relative abundance of rhizospheric functional bacteria of STD and STE under control or NaCl treatments.

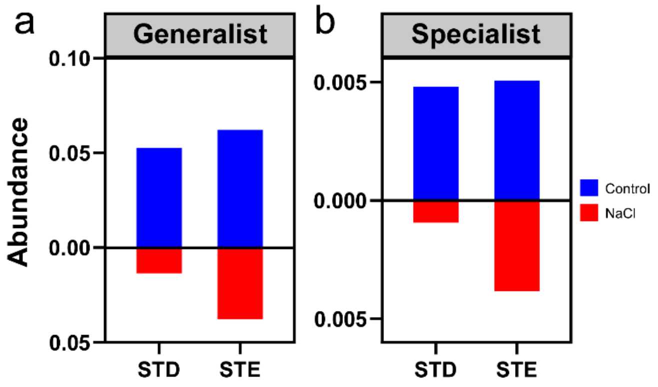

Fig. S5. Bacterial ecological strategy analyses of the bulk microbes of STD and STE under normal or saline conditions. Relative abundance of rhizospheric generalist (a) or specialist (b) bacteria of STD and STE under control or NaCl treatments.

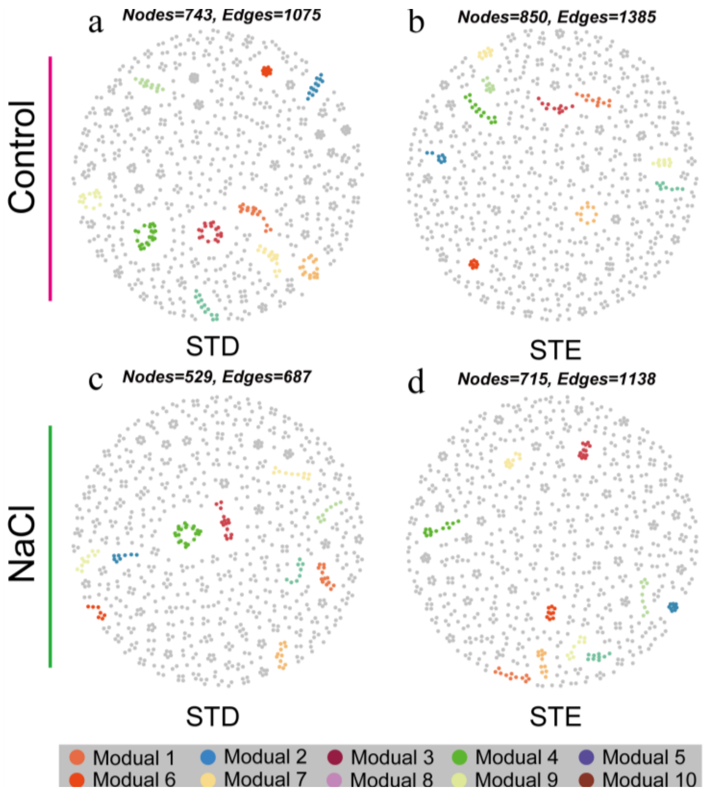

Fig. S6. Network patterns in the bulk soil between rice plants. Visualization of constructed networks in STD and STE under control (a-b) and NaCl treatment (c-d). Different modules are shown in different colors.

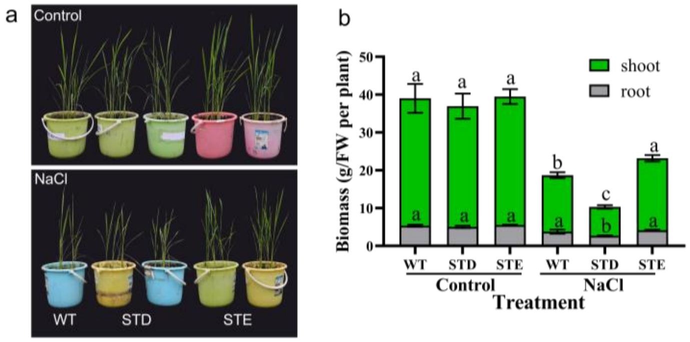

Fig. S7. Bacterial change contributes to the phenotype of rice under saline conditions. Photographs (a) and fresh weight (b) showing bacterial effects on rice growth due to C deposition differences. The means and standard deviation of duplicate samples were plotted (n = 6). Mean values with different letters were significantly different ($P < 0.05$).
