# Supplementary material for: Enhancing Photosynthetic Carbon Transport in Rice Plant Optimizes Rhizosphere Bacterial Community in Saline Soil
Source: Int J Mol Sci. 2024 Nov 13;25(22):12184. doi: 10.3390/ijms252212184 (PMC11594718; doi:10.3390/ijms252212184)
Supplement: Supplementary file 1 [file ijms-25-12184-s001.zip › Supplementary Tables.pdf]

Supplementary Tables

Table S1. Basic properties of soil for pot experiments.

pH	Soil organic matter	NH_4^+-N	NO_3^--N	Available P	Available K	Na^+
Soil:H_2O(1:2.5)	(g·kg^{-1})	(mg·kg^{-1})	(mg·kg^{-1})	(mg·kg^{-1})	(mg·kg^{-1})	(mg·kg^{-1})
6.5	16.1	26.19	10.51	17.1	269.55	ND

(ND, Not detectable)

Table S2. Primers and procedures of soil microbe analysis in real-time PCR.

Target microbes	Primers（5´-3´）	PCR procedure
OsSUT5	F, TTGCCATTGTTGTCCCACAG<br>R, ACTCCACAGATGAAGGCGAA	An initial cycle of 30 sec at 95 °C; 40 cycles of 10sec at 95 °C, 30 sec at 55 °C, and 30 sec at 72 °C; 15 sec at 95 °C, 60 sec at 60 °C, and 15 sec at 95 °C for melt curve.
OsActin	F, TGGTCGTACCACAGGTATTGTGTT<br>R, AAGGTCGAGACGAAGGATAGCAT	An initial cycle of 30 sec at 95 °C; 40 cycles of 10sec at 95 °C, 30 sec at 55 °C, and 30 sec at 72 °C; 15 sec at 95 °C, 60 sec at 60 °C, and 15 sec at 95 °C for melt curve.
Bacteria	515F, GTGCCAGCMGCCGCGGTAA<br>907R, CCGTCAATTCCTTTGAGTTT	An initial cycle of 30 sec at 95 °C; 40 cycles of 10sec at 95 °C, 30 sec at 55 °C, and 30 sec at 72 °C; 15 sec at 95 °C, 60 sec at 60 °C, and 15 sec at 95 °C for melt curve.

Table S3. Information on the physical and chemical properties of three types of rice plants under different treatments.

Control	NH_4^+-N	AP	AK	NO_3^--N	TN	TP	TK
WT	27.8+2.92a	33.66+0.48b	31.35+1.23a	12.77+0.07a	1.25+0.58a	0.33+0.01a	9.97+0.22a
STD	27.2+2.35a	34.26+1.28a	31.92+1.24a	12.82+0.13a	1.31+0.01a	0.33+0.01a	9.87+0.21a
STE	27.86+0.78a	33.36+1.17a	31.28+0.92a	12.86+0.09a	1.23+0.04a	0.33+0.01a	9.99+0.09a

NaCl	NH_4^+-N	AP	AK	NO_3^--N	TN	TP	TK
WT	24.4+0.74b	32.76+2.36b	24.4+1.28b	12.96+0.11a	1.09+0.06a	0.33+0.01a	9.71+0.06a
STD	32.33+1.22a	38..33+0.64a	28.3+1.24a	12.9+0.08a	1.24+0.03a	0.36+0.01a	10.01+0.15a
STE	11.28+1.35c	21.32+0.96c	18.25+1.24c	13.05+0.17a	1.02+0.47a	0.31+0.01a	9.67+0.05a

Table S4. Rhizosphere microbial information of DAA.

STE vs. STD under Control

Enriched	Phylum	Functional/Pathogenic	Generalist/Specialist
ASV36759	Halanaerobiaeota	F	Un
ASV48	Firmicutes	F	S
ASV6877	Halanaerobiaeota	F	Un
ASV162	Bacteroidota	F	G
ASV3060	Actinobacteriota	F	S
ASV106	Firmicutes	F	S
ASV2600	Bacteria	Unclassified	Un
ASV63	Bacteroidota	F	G
ASV2630	Firmicutes	F	S
ASV266	Firmicutes	F	S
ASV73	Firmicutes	F	S
ASV496	Bdellovibrionota	F	S
ASV31297	Bacteria	Unclassified	Un
ASV189	Firmicutes	F	S
ASV172	Firmicutes	F	S
ASV2625	Bacteroidota	F	G
ASV16182	Actinobacteriota	F	S
ASV3	Bacteroidota	F	G
ASV2555	Desulfobacterota	F	S
ASV207	Bacteroidota	F	G
ASV942	Chloroflexi	F	G
ASV661	Chloroflexi	F	G
ASV12	Firmicutes	F	S
ASV36785	Firmicutes	F	S
ASV5469	Firmicutes	F	S
ASV36840	Firmicutes	F	S

ASV891	Firmicutes	F	S
ASV3315	Planctomycetota	F	G
ASV16165	Firmicutes	F	S
ASV2552	Bacteroidota	F	G
ASV9935	Actinobacteriota	F	S
ASV9607	Chloroflexi	F	G
ASV4682	Firmicutes	F	S
ASV7382	Chloroflexi	F	G
ASV514	Bacteroidota	F	G
ASV2812	Cyanobacteria	F	S
ASV281	Desulfobacterota	F	S
ASV3323	Patescibacteria	F	S
ASV653	Chloroflexi	F	G
ASV7102	Bacteroidota	F	G
ASV30391	Cyanobacteria	F	S
ASV2538	Desulfobacterota	F	S
ASV474	Firmicutes	F	S
ASV2050	Firmicutes	F	S
ASV6864	Halanaerobiaeota	F	Un
ASV6936	Armatimonadota	F	S
ASV31296	Armatimonadota	F	S
ASV29931	Armatimonadota	F	S
ASV557	Actinobacteriota	F	S
ASV16621	Proteobacteria	F	G
ASV966	Spirochaetota	F	S
ASV6959	Acidobacteriota	F	G
ASV509	Firmicutes	F	S
ASV493	Myxococcota	F	G
ASV1119	Armatimonadota	F	S
ASV1089	Chloroflexi	F	G
ASV160	Firmicutes	F	S
ASV7508	Verrucomicrobiota	F	G
ASV5123	Actinobacteriota	F	S
ASV7288	WS1	Unclassified	Un
ASV746	Chloroflexi	F	G
ASV2824	Firmicutes	F	S

Depleted	Phylum	Functional/Pathogenic	Generalist/Specialist
ASV4653	Firmicutes	F	S
ASV6668	Proteobacteria	F	G
ASV3061	Acidobacteriota	F	G
ASV3047	Chloroflexi	F	G

ASV7540	Acidobacteriota	F	G
ASV51764	Desulfobacterota	F	S
ASV10258	Gemmatimonadota	F	G
ASV12488	Bacteroidota	F	G
ASV12419	Bacteroidota	F	G
ASV3541	Planctomycetota	F	G
ASV579	Bacteroidota	F	G
ASV5138	Acidobacteriota	F	G
ASV2821	Chloroflexi	F	G
ASV12528	Firmicutes	F	S
ASV6928	Nitrospirota	F	G
ASV7081	Chloroflexi	F	G
ASV23330	Firmicutes	F	S
ASV4695	Desulfobacterota	F	S
ASV2953	Chloroflexi	F	G
ASV12413	Bacteroidota	F	G
ASV12743	Entotheonellaeota	P	S
ASV4727	Bacteroidota	F	G
ASV11456	Desulfobacterota	F	S
ASV12562	Bacteroidota	F	G
ASV7335	Myxococcota	F	G
ASV7479	Acidobacteriota	F	G
ASV4913	Chloroflexi	F	G
ASV12878	Chloroflexi	F	G
ASV6105	WS2	Unclassified	Un
ASV692	Chloroflexi	F	G
ASV12714	Nitrospirota	F	G
ASV129	Bacteroidota	F	G
ASV12570	Sva0485	Unclassified	Un
ASV39122	Proteobacteria	F	G
ASV7364	Bacteroidota	F	G
ASV12532	Cyanobacteria	F	S
ASV3796	Planctomycetota	F	G

STE vs. STD under NaCl

Enriched	Phylum	Functional/Pathogenic	Generalist/Specialist
ASV418	Firmicutes	F	S
ASV11053	Verrucomicrobiota	F	G
ASV7314	Proteobacteria	F	G
ASV11427	Planctomycetota	F	G
ASV9846	Chloroflexi	F	G
ASV2820	Proteobacteria	F	G
ASV2532	Bacteroidota	F	G

ASV2746	Bacteroidota	F	G
ASV2846	Cyanobacteria	F	S
ASV7225	Planctomycetota	F	G
ASV48	Firmicutes	F	S
ASV4012	Chloroflexi	F	G
ASV41	Desulfobacterota	F	S
ASV2562	Acidobacteriota	F	G
ASV2660	Acidobacteriota	F	G
ASV474	Firmicutes	F	S
ASV3107	Desulfobacterota	F	S
ASV2758	Bacteroidota	F	G
ASV9307	Chloroflexi	F	G
ASV3736	Chloroflexi	F	G
ASV775	Bacteroidota	F	G
ASV3219	Desulfobacterota	F	S
ASV410	Proteobacteria	F	G
ASV641	Bacteroidota	F	G
ASV20146	Desulfobacterota	F	S
ASV12519	Proteobacteria	F	G
ASV4917	Planctomycetota	F	G
ASV7856	Planctomycetota	F	G
ASV3551	Gemmatimonadota	F	G
ASV30006	Actinobacteriota	F	S
ASV730	Firmicutes	F	S
ASV76220	Acidobacteriota	F	G
ASV38939	Bacteroidota	F	G
ASV7253	Myxococcota	F	G
ASV14365	Planctomycetota	F	G
ASV4757	Myxococcota	F	G
ASV112	Myxococcota	F	G
ASV65607	Proteobacteria	F	G
ASV18728	Planctomycetota	F	G
ASV497	Proteobacteria	F	G
ASV16182	Actinobacteriota	F	S
ASV4988	Methylomirabilota	F	G
ASV689	Firmicutes	F	S
ASV3	Bacteroidota	F	G
ASV11046	Acidobacteriota	F	G
ASV39095	Sva0485	Unclassified	Un
ASV6668	Proteobacteria	F	G
ASV7045	Acidobacteriota	F	G
ASV2576	Proteobacteria	F	G
ASV18314	Latescibacterota	F	G

ASV15301	Gemmatimonadota	F	G
ASV11298	Patescibacteria	F	S
ASV3423	Chloroflexi	F	G
ASV22429	Actinobacteriota	F	S
ASV2573	Firmicutes	F	S
ASV6994	Myxococcota	F	G
ASV25886	Planctomycetota	F	G
ASV7150	Actinobacteriota	F	S
ASV26786	Planctomycetota	F	G
ASV14711	Methylomirabilota	F	G
ASV10894	Proteobacteria	F	G
ASV21568	Planctomycetota	F	G
ASV6944	Chloroflexi	F	G
ASV9526	Methylomirabilota	F	G
ASV231	Firmicutes	F	S
ASV979	Chloroflexi	F	G
ASV6925	Actinobacteriota	F	S
ASV16174	Armatimonadota	F	S
ASV16457	Desulfobacterota	F	S
ASV62725	Acidobacteriota	F	G
ASV4759	Actinobacteriota	F	S
ASV739	Desulfobacterota	F	S
ASV7784	Sumerlaeota	F	G
ASV4874	Chloroflexi	F	G
ASV42125	Planctomycetota	F	G
ASV674	Proteobacteria	F	G
ASV14320	Bacteroidota	F	G
ASV9508	Planctomycetota	F	G
ASV18624	Bacteroidota	F	G
ASV106	Firmicutes	F	S
ASV1091	Chloroflexi	F	G
ASV5234	Acidobacteriota	F	G
ASV4746	Firmicutes	F	S
ASV11184	Firmicutes	F	S
ASV12575	Chloroflexi	F	G
ASV13229	Chloroflexi	F	G
ASV3548	Planctomycetota	F	G
ASV11484	Acidobacteriota	F	G
ASV595	Chloroflexi	F	G
ASV373	Chloroflexi	F	G
ASV2260	Chloroflexi	F	G
ASV234	Actinobacteriota	F	S
ASV5102	Proteobacteria	F	G

ASV2798	Chloroflexi	F	G
ASV3417	Zixibacteria	F	S
ASV2702	Myxococcota	F	G
ASV419	Bdellovibrionota	F	S
ASV4704	Proteobacteria	F	G
ASV7512	Chloroflexi	F	G
ASV7139	Bacteroidota	F	G
ASV589	Actinobacteriota	F	S
ASV995	Chloroflexi	F	G
ASV2754	Actinobacteriota	F	S
ASV2679	Firmicutes	F	S
ASV868	Firmicutes	F	S
ASV2876	Nitrospirota	F	G
ASV33104	Desulfobacterota	F	S
ASV5018	Chloroflexi	F	G
ASV68404	Acidobacteriota	F	G
ASV23658	Planctomycetota	F	G
ASV14339	Bacteroidota	F	G
ASV28451	Chloroflexi	F	G
ASV2575	Acidobacteriota	F	G
ASV4773	Actinobacteriota	F	S
ASV12563	Actinobacteriota	F	S
ASV4679	Myxococcota	F	G
ASV238	Bacteroidota	F	G
ASV649	Chloroflexi	F	G
ASV11101	Acidobacteriota	F	G
ASV18344	Planctomycetota	F	G
ASV7245	Chloroflexi	F	G
ASV2649	Desulfobacterota	F	S
ASV7884	Armatimonadota	F	S
ASV18350	Deferrisomatota	F	S
ASV12793	Planctomycetota	F	G
ASV974	Chloroflexi	F	G
ASV480	Desulfobacterota	F	S
ASV566	Actinobacteriota	F	S
ASV109	Actinobacteriota	F	S
ASV766	Chloroflexi	F	G
ASV865	Chloroflexi	F	G
ASV1015	Chloroflexi	F	G
ASV4	Firmicutes	F	S
ASV2538	Desulfobacterota	F	S
ASV241	Actinobacteriota	F	S
ASV656	Proteobacteria	F	G
ASV723	Planctomycetota	F	G

ASV10914	Bacteroidota	F	G
ASV2731	Deferrisomatota	F	S
ASV26627	Planctomycetota	F	G
ASV12	Firmicutes	F	S
ASV175	Firmicutes	F	S
ASV1067	Planctomycetota	F	G
ASV249	MBNT15	F	S
ASV1118	Proteobacteria	F	G
ASV12513	Acidobacteriota	F	G
ASV50	Firmicutes	F	S
ASV2561	Proteobacteria	F	G
ASV92	Firmicutes	F	S
ASV11006	Chloroflexi	F	G
ASV23274	Acidobacteriota	F	G
ASV11135	Acidobacteriota	F	G
ASV2849	Proteobacteria	F	G
ASV541	Proteobacteria	F	G
ASV152	Bacteroidota	F	G
ASV4901	Proteobacteria	F	G
ASV780	Chloroflexi	F	G
ASV703	Chloroflexi	F	G
ASV622	Planctomycetota	F	G
ASV337	Methylomirabilota	F	G
ASV825	Proteobacteria	F	G
ASV3249	Planctomycetota	F	G
ASV140	Firmicutes	F	S
ASV2942	Chloroflexi	F	G

Depleted	Phylum	Functional/Pathogenic	Generalist/Specialist
ASV687	Proteobacteria	F	G
ASV431	Actinobacteriota	F	S
ASV25290	Myxococcota	F	G
ASV12456	Proteobacteria	F	G
ASV759	Proteobacteria	F	G
ASV2993	Proteobacteria	F	G
ASV4993	Bacteria	Unclassified	Unclassified
ASV14351	Proteobacteria	F	G
ASV6948	Bacteroidota	F	G
ASV12474	Firmicutes	F	S
ASV7017	Patescibacteria	F	S
ASV286	Chloroflexi	F	G
ASV12510	Bacteroidota	F	G
ASV5237	Bacteroidota	F	G

ASV2609	Proteobacteria	F	G
ASV321	Actinobacteriota	F	S
ASV7626	Chloroflexi	F	G
ASV784	Myxococcota	F	G
ASV618	Firmicutes	F	S
ASV6946	Bacteroidota	F	G
ASV6975	Chloroflexi	F	G
ASV585	Chloroflexi	F	G
ASV18398	Firmicutes	F	S
ASV829	Proteobacteria	F	G
ASV874	Firmicutes	F	S
ASV3069	Chloroflexi	F	G
ASV3279	Chloroflexi	F	G
ASV1324	Proteobacteria	F	G
ASV764	Sumerlaeota	F	G
ASV80	Desulfobacterota	F	S
ASV3809	Cyanobacteria	F	S
ASV15167	Actinobacteriota	F	S
ASV314	Firmicutes	F	S
ASV1190	Chloroflexi	F	G
ASV5110	Chloroflexi	F	G
ASV955	Chloroflexi	F	G
ASV16827	Proteobacteria	F	G
ASV23330	Firmicutes	F	S
ASV23228	Firmicutes	F	S
ASV12588	Bacteroidota	F	G
ASV77713	Firmicutes	F	S
ASV7059	Firmicutes	F	S
ASV63	Bacteroidota	F	G
ASV14606	Chloroflexi	F	G
ASV4672	Bacteria	Unclassified	Unclassified
ASV6905	Actinobacteriota	F	S
ASV927	Chloroflexi	F	G
ASV11055	WS1	Unclassified	Un
ASV12455	Proteobacteria	F	G
ASV12446	Bacteria	Unclassified	Unclassified
ASV16127	Actinobacteriota	F	S
ASV16205	Bacteria	Unclassified	Unclassified
ASV12540	Chloroflexi	F	G
ASV3414	Verrucomicrobiota	F	G
ASV24949	Chloroflexi	F	G
ASV4994	Chloroflexi	F	G
ASV16856	Chloroflexi	F	G
ASV18772	Patescibacteria	F	S

ASV23337	Firmicutes	F	S
ASV9437	Proteobacteria	F	G
ASV36795	Bacteria	Unclassified	Unclassified
ASV12720	Patescibacteria	F	S
ASV5063	Verrucomicrobiota	F	G
ASV14429	Planctomycetota	F	G
ASV3056	WS4	Unclassified	Un
ASV2815	Bacteroidota	F	G
ASV51828	Gemmatimonadota	F	G
ASV14306	Myxococcota	F	G
ASV20008	Firmicutes	F	S
ASV12633	Proteobacteria	F	G
ASV14348	Proteobacteria	F	G
ASV7915	Firmicutes	F	S
ASV16590	Proteobacteria	F	G
ASV2605	Actinobacteriota	F	S
ASV1001	Chloroflexi	F	G
ASV16150	Actinobacteriota	F	S
ASV6891	Desulfobacterota	F	S
ASV2913	Proteobacteria	F	G
ASV2686	Chloroflexi	F	G
ASV9432	Chloroflexi	F	G
ASV1181	Firmicutes	F	S
ASV7224	Firmicutes	F	S
ASV1058	Chloroflexi	F	G
ASV20223	Bacteroidota	F	G
ASV17980	Acidobacteriota	F	G
ASV2971	Hydrogenedentes	F	G
ASV55908	Proteobacteria	F	G
ASV933	Gemmatimonadota	F	G
ASV4893	Proteobacteria	F	G
ASV18561	Chloroflexi	F	G
ASV3534	Proteobacteria	F	G
ASV8515	Chloroflexi	F	G
ASV11097	Chloroflexi	F	G
ASV12445	Proteobacteria	F	G
ASV7506	Planctomycetota	F	G
ASV1802	Actinobacteriota	F	S
ASV848	Proteobacteria	F	G
ASV16613	Chloroflexi	F	G
ASV1186	Methylomirabilota	F	G
ASV2730	Firmicutes	F	S

ASV20282	Bacteroidota	F	G
ASV16783	Cyanobacteria	F	S
ASV22371	Actinobacteriota	F	S
ASV2531	Armatimonadota	F	S
ASV39122	Proteobacteria	F	G
ASV55866	Proteobacteria	F	G
ASV12496	Bacteroidota	F	G
ASV55071	Proteobacteria	F	G
ASV4813	Acidobacteriota	F	G
ASV56514	Bacteria	Unclassified	Unclassified
ASV12558	Firmicutes	F	S
ASV23282	Proteobacteria	F	G
ASV6878	Patescibacteria	F	S
ASV36768	Firmicutes	F	S
ASV5050	Cyanobacteria	F	S

Table S5. Classification of functional/pathogen bacteria.

Functional bacteria	References	Pathogen bacteria	References	Unclassified
Methylomirabilota	1	Entotheonellaeota	26	WS4
Latescibacterota	2			Norank
Sumerlaeota	3			WS1
Deferrisomatota	4			WS2
MBNT15	5			Sva0485
Hydrogenedentes	6			
Zixibacteria	7			
Firmicutes	8			
Bacteroidota	9			
Actinobacteriota	10			
Bdellovibrionota	11			
Desulfobacterota	12			
Chloroflexi	13			
Planctomycetota	14			
Cyanobacteria	15			
Proteobacteria	16			
Spirochaetota	17			
Acidobacteriota	18			
Verrucomicrobiota	19			
Gemmatimonadota	20			
Nitrospirota	21			
Myxococcota	22			
Halanaerobiaeota	23			
Patescibacteria	24			
Armatimonadota	25			

1. Hayat R, Ali S, Amara U, Khalid R, Ahmed I. 2010. Soil beneficial bacteria and their role in plant growth promotion: a review. 4. Ann Microbiol 60:579–598.
2. Lan J, Wang S, Wang J, Qi X, Long Q, Huang M. 2022. The Shift of Soil Bacterial Community After Afforestation Influence Soil Organic Carbon and Aggregate Stability in Karst Region. Front Microbiol 13.
3. Fang Y, Yuan Y, Liu J, Wu G, Yang J, Hua Z, Han J, Zhang X, Li W, Jiang H. 2021. Casting Light on the Adaptation Mechanisms and Evolutionary History of the Widespread Sumerlaeota. mBio 12:10.1128/mbio.00350-21.
4. Ye Y-L, Ma K-J, Fu Y-H, Wu Z-C, Fu G-Y, Sun C, Xu X-W. 2023. The heterogeneity of microbial diversity and its drivers in two types of sediments from tidal flats in Beibu Gulf, China. Front Mar Sci 10.
5. Begmatov S, Beletsky AV, Dedysh SN, Mardanov AV, Ravin NV. 2022. Genome analysis of the candidate phylum MBNT15 bacterium from a boreal peatland predicted its respiratory versatility and dissimilatory iron metabolism. Front Microbiol 13.
6. Zahid MS, Hussain M, Song Y, Li J, Guo D, Li X, Song S, Wang L, Xu W, Wang S. 2022. Root-Zone Restriction Regulates Soil Factors and Bacterial Community Assembly of Grapevine. 24. Int J Mol Sci 23:15628.
7. Sun C, Wang R, Tang G, Cai S, Shi H, Liu F, Xie H, Zhu J, Xiong Q. 2023. Integrated 16S and metabolomics revealed the mechanism of drought resistance and nitrogen uptake in rice at the heading stage under different nitrogen levels. Front Plant Sci 14.
8. Wieczorek AS, Schmidt O, Chatzinotas A, von Bergen M, Gorissen A, Kolb S. 2019. Ecological Functions of Agricultural Soil Bacteria and Microeukaryotes in Chitin Degradation: A Case Study. Front Microbiol 10.
9. Huang J, Gao K, Yang L, Lu Y. 2023. Successional action of Bacteroidota and Firmicutes in decomposing straw polymers in a paddy soil. Environ Microbiome 18:76.
10. Liang X, Ye J, Xue Y, Aili T, Han S, Zhang J, Meng S, Aimaiti R, Zhang M, Jia Z, Gomiero A, Wang W, Yang J. 2024. Microplastics and their interaction with

microorganisms in Bosten Lake sediment. Water Res 261:122060.

11. Waso M, Reyneke B, Havenga B, Khan S, Khan W. 2021. Insights into Bdellovibrio spp. mechanisms of action and potential applications. World J Microbiol Biotechnol 37:85.
12. Masuda Y, Mise K, Xu Z, Zhang Z, Shiratori Y, Senoo K, Itoh H. 2024. Global soil metagenomics reveals distribution and predominance of Deltaproteobacteria in nitrogen-fixing microbiome. Microbiome 12:95.
13. Speirs LBM, Rice DTF, Petrovski S, Seviour RJ. 2019. The Phylogeny, Biodiversity, and Ecology of the Chloroflexi in Activated Sludge. Front Microbiol 10.
14. Vitorino IR, Lage OM. 2022. The Planctomycetia: an overview of the currently largest class within the phylum Planctomycetes. Antonie Van Leeuwenhoek 115:169–201.
15. Roque J, Brito Â, Rocha M, Pissarra J, Nunes T, Bessa M, Vieira J, Vieira CP, Melo P, Tamagnini P. 2023. Isolation and characterization of soil cyanobacteria and microalgae and evaluation of their potential as plant biostimulants. Plant Soil 493:115–136.
16. Kersters K, De Vos P, Gillis M, Swings J, Vandamme P, Stackebrandt E. 2006. Introduction to the Proteobacteria, p. 3–37. In Dworkin, M, Falkow, S, Rosenberg, E, Schleifer, K-H, Stackebrandt, E (eds.), The Prokaryotes: Volume 5: Proteobacteria: Alpha and Beta Subclasses. Springer, New York, NY.
17. Svartström O, Alneberg J, Terrapon N, Lombard V, de Bruijn I, Malmsten J, Dalin A-M, EL Muller E, Shah P, Wilmes P, Henrissat B, Aspeborg H, Andersson AF. 2017. Ninety-nine de novo assembled genomes from the moose (Alces alces) rumen microbiome provide new insights into microbial plant biomass degradation. ISME J 11:2538–2551.
18. Kalam S, Basu A, Ahmad I, Sayyed RZ, El-Enshasy HA, Dailin DJ, Suriani NL. 2020. Recent Understanding of Soil Acidobacteria and Their Ecological Significance: A Critical Review. Front Microbiol 11.
19. Baliyarsingh B, Dash B, Nayak S, Nayak SK. 2022. Soil Verrucomicrobia and

Their Role in Sustainable Agriculture, p. 105–124. In Nayak, SK, Baliyarsingh, B, Mannazzu, I, Singh, A, Mishra, BB (eds.), Advances in Agricultural and Industrial Microbiology: Volume 1: Microbial Diversity and Application in Agroindustry. Springer Nature, Singapore.

20. Mujakić I, Piwosz K, Koblížek M. 2022. Phylum Gemmatimonadota and Its Role in the Environment. 1. Microorganisms 10:151.
21. Palomo A, Dechesne A, Pedersen AG, Smets BF. 2022. Genomic profiling of Nitrospira species reveals ecological success of comammox Nitrospira. Microbiome 10:204.
22. Zou D, Zhang C, Liu Y, Li M. 2024. Biogeographical distribution and community assembly of Myxococcota in mangrove sediments. Environ Microbiome 19:47.
23. Roush DW, Elias DA, Mormile MR. Metabolic Capabilities of the Members of the Order Halanaerobiales and Their Potential Biotechnological Applications. Curr Biotechnol 3:3–9.
24. Zhang Z, Wan J, Ye G, Wang Y, Bai Y, Yan Z. 2025. Effects of salinity and betaine addition on anaerobic granular sludge properties and microbial community succession patterns in organic saline wastewater. J Environ Sci 147:310–321.
25. Liew KJ, Shahar S, Shamsir MS, Shaharuddin NB, Liang CH, Chan K-G, Pointing SB, Sani RK, Goh KM. 2024. Integrating multi-platform assembly to recover MAGs from hot spring biofilms: insights into microbial diversity, biofilm formation, and carbohydrate degradation. Environ Microbiome 19:29.
26. Zhang Y, Sun X, Qian C, Li L, Shang X, Xiao X, Gao Y. 2022. Impact of Petroleum Contamination on the Structure of Saline Soil Bacterial Communities. Curr Microbiol 79:351.

Table S6. Network patterns in the Rhizosphere and bulk between rice plants.

Rhizosphere		WT	STD	STE
Control	Positive	66.50%	69.12%	61.37%
	Negative	33.50%	30.88%	38.63%
NaCl	Positive	60.33%	63.35%	62.83%
	Negative	39.67%	36.65%	37.17%
Bulk		WT	STD	STE
Control	Positive	72.70%	73.40%	71.14%
	Negative	27.30%	26.60%	28.86%
NaCl	Positive	77.00%	77.82%	74.68%
	Negative	23.00%	22.18%	25.32%
